# 20-hydroxyeiscosatetraenoic acid may be as a predictor of malignant middle cerebral artery infarction in patients with massive middle cerebral artery infarction

**DOI:** 10.1186/s12883-021-02456-6

**Published:** 2021-11-09

**Authors:** Xingyang Yi, Qiang Zhou, Ting Qing, Bing Ming, Jing Lin, Jie Li, Jie Lin

**Affiliations:** 1 Department of Neurology, People’s Hospital of Deyang City, Deyang, 618000 Sichuan China; 2 Department of Neurology, the Third Affiliated Hospital of Wenzhou Medical University, Wenzhou, 325200 Zhejiang China; 3 Department of Radiology, People’s Hospital of Deyang City, Deyang, 618000 Sichuan China; 4 Department of PET/CT, the First Affiliated Hospital of Wenzhou Medical University, Wenzhou, 325000 Zhejiang China

**Keywords:** 20-hydroxyeicosatetraenoic acid, Brain edema, Malignant middle cerebral artery infarction, Massive middle cerebral artery infarction, Prognosis

## Abstract

**Background:**

Early identification of massive middle cerebral artery infarction (MCAI) at risk for malignant MCAI (m-MCAI) may be useful in selecting patients for aggressive therapies. The aim of this study was to determine whether CYP metabolites may help to predict impending m-MCAI.

**Methods:**

This is a prospective, two-center observational study in 256 patients with acute massive MCAI. Plasma levels of 20-hydroxyeicosatetraenoic acid (20-HETE), epoxyeicosatrienoic acids, and dihydroxyeicosatrienoic acids were measured at admission. Brain computed tomography (CT) was performed at admission and repeated between day 3 and 7, or earlier if there was neurological deterioration. The primary outcome was m-MCAI. The m-MCAI was diagnosed when follow-up brain CT detected a more than two-thirds space-occupying MCAI with midline shift, compression of the basal cisterns, and neurological worsening.

**Results:**

In total of 256 enrolled patients, 77 (30.1%) patients developed m-MCAI. Among the 77 patients with m-MCAI, 60 (77.9%) patients died during 3 months of stroke onset. 20-HETE level on admission was significantly higher in patients with m-MCAI than those without m-MCAI. There was an increase in the risk of m-MCAI with increase of 20-HETE levels. The third and fourth quartiles of 20-HETE levels were independent predictors of m-MCAI (OR: 2.86; 95% CI: 1.16 – 6.68; *P =* 0.025, and OR: 4.23; 95% CI: 1.35 – 8.26; *P =* 0.002, respectively).

**Conclusions:**

Incidence of m-MCAI was high in patients with massive MCAI and the prognosis of m-MCAI is very poor. Elevated plasma 20-HETE may be as a predictor for m-MCAI in acute massive MCAI, and it might useful in clinical practice in therapeutic decision making.

## Background

Massive middle cerebral artery infarction (MCAI) accounts for 10 to 15% of all stroke patients, which usually caused by acute occlusion of internal carotid artery or the proximal middle cerebral artery (MCA) [1, 2], and of these patients, malignant MCAI (m-MCAI) reaches 30 to 50% [2–4]. The m-MCAI is the most devastating form of ischemic stroke, is characterized with large supratentorial infarcts and space-occupying brain edema followed by cerebral herniation [2, 5]. The mortality is approximately 80% in m-MCAI patients, and those patients who survive experience severe disabilities [6, 7]. Decompressive hemicraniectomy (DHC) is recommended when it is performed within 48 h after stroke onset in patients with m-MCAI [8, 9]. Therefore, early identification or prediction of m-MCAI is very essential for timely application of DHC in patients with massive MCAI.

It is noted that not all patients with massive MCAI would develop m-MCAI [2, 4, 10–12]. Therefore, identification of key mechanism involved in m-MCAI and predictor for m-MCAI will be of important significance for the early diagnosis and treatment of m-MCAI among patients with massive MCAI. For the last two decades, prediction of m-MCAI using radiological variables, clinical risk factors, and molecular markers has been thoroughly investigated [2–4, 8, 13, 14]. However, the underlying basic mechanisms and predictors for m-MCAI are not completely understood.

The loss of integrity of the endothelial basal lamina and blood-brain barrier (BBB) disruption are believed to be the important cause of edema after focal cerebral ischemia. Arachidonic acid (AA) is a membrane fatty acid that can be metabolized into 20-hydroxyeicosatetraenoic acid (20-HETE) and epoxyeicosatrienoic acids (EETs) by cytochrome P450 (CYP) ω-hydroxylase and CYP epoxygenases, respectively, and EETs are then metabolized to yield less biologically active dihydroxyeicosatrienoic acids (DiHETEs) by soluble epoxide hydrolase (sEH) [15]. 20-HETE and EETs have inflammatory activities [16]. Meanwhile, 20-HETE, as a potent vasoconstrictor, has been shown in the cerebral vasculature of the stroke-prone spontaneously hypertensive rat and contributes to stroke severity [17]. EETs play an important role in neuroprotection and cerebral blood flow regulation after brain injury [18]. Our previous studies demonstrated that CYP metabolite levels were associated with early neurologic deterioration and functional outcome, and may be as a predictor for early neurologic deterioration in acute ischemic stroke [19, 20]. However, although considerable advances have been made, the association of CYP metabolites with m-MCAI development has not been definitely determined.

In this study, we hypothesized that levels of early CYP metabolites, including 20-HETE, EETs and DiHETEs could predict m-MCAI. Therefore, we conducted a prospective, two-center observational study that aimed to explore potential CYP metabolites involved in the m-MCAI and to elucidate their possible mechanisms in m-MCAI development.

## Materials and methods

### Study population

This study was conducted in People’s Hospital of Deyang City, the First Affiliated Hospital of Wenzhou Medical University and the Third Affiliated Hospital of Wenzhou Medical University. The study protocol was approved by the Ethics Committee at the participating hospitals. Written informed consent was obtained from each patient prior to study enrollment.

**Fig. 1:**
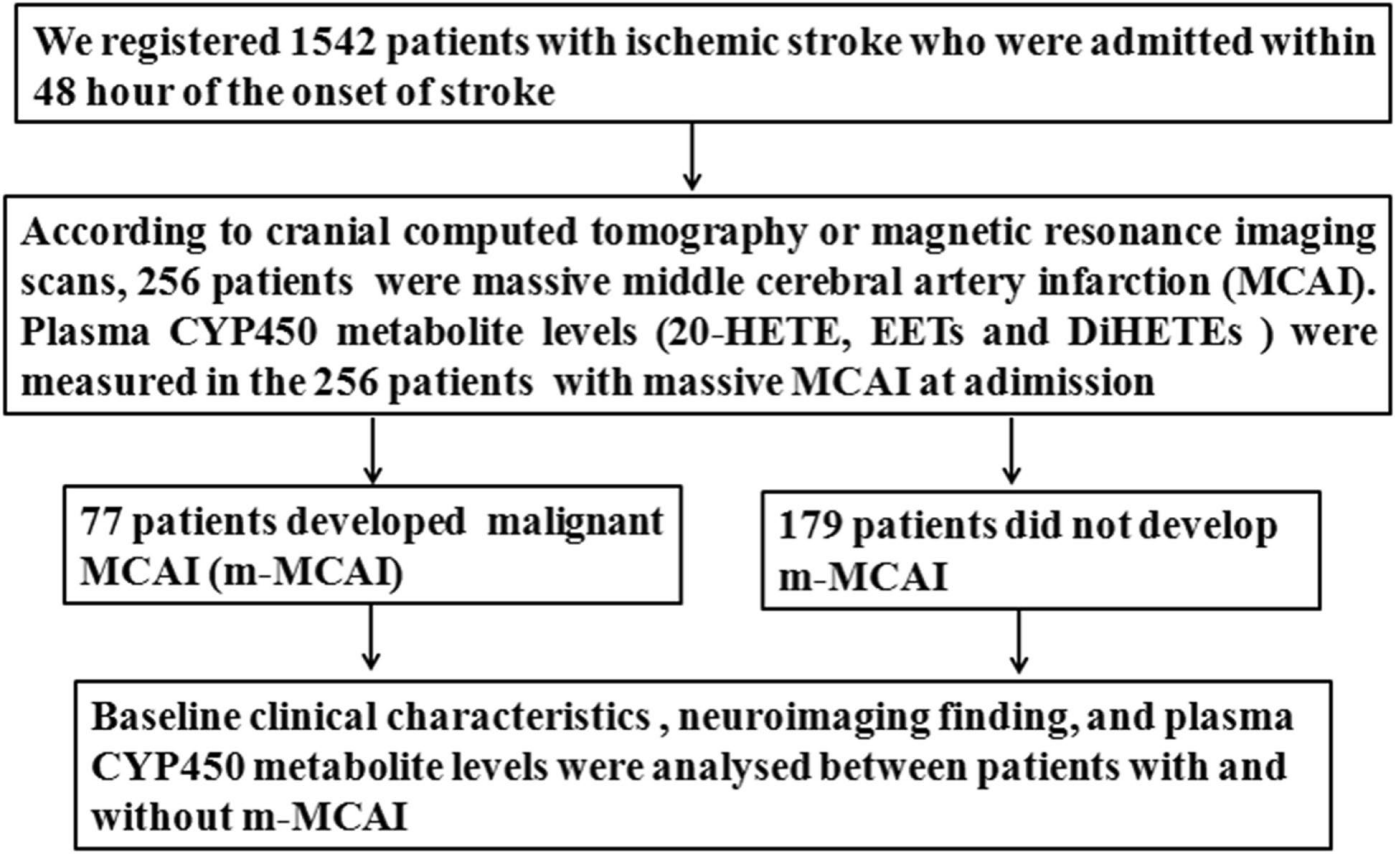
Flowchart in this study

Between October 2011 and September 2014, we consecutively registered 1542 patients who had suffered their first-ever stroke and were admitted to the participating hospitals. Data were recorded at the time of assessment using a standardized structured form. Detailed methods for data collection of the patients were described in our previous articles [14, 21]. In this study, we enrolled the patients who were suffered from massive MCAI and admitted to the participating hospitals within 48 h of their index stroke onset. Massive MCAI was defined as > 50% of the MCA territory on early cranial computed tomography (CT) or magnetic resonance imaging (MRI) scans, with or without the involvement of the adjacent territories [14, 22]. The patients with incomplete hospital records or missing imaging, preexisting score of more than 2 on the modified Rankin scale (mRS), participating in other clinical trials or receiving thrombectomy, and unwilling to participate in this study were excluded. According to aforementioned inclusion criteria and exclusion criteria, a total of 256 patients with massive MCA infarction were enrolled, the detailed data cleaning procedure was presented in Fig. 1.

All enrolled patients had an initial brain CT scan at admission. Early signs of cerebral infarction on CT included: (1) the presence of focal hypodensity consistent with the clinical findings,obscuration of the lenticular nucleus or the cortex; (2) mass effect was determined by grading hemispheric swelling [23]: effacement of the cortical sulci (grade I), ventricular asymmetry grade II), shifting of the structures of the median line (grade III) [23]. For the extent of MCAI, Alberta Stroke Program Early CT Score (ASPECTS) was evaluated in the first CT examination [24]. A second CT or MRI was performed between 3 days and 7 days of hospitalization to measure the infarct volume. The infarct volume was determined using the Coniglobus formula 0.5 × *a* × *b* × *c* (where *a* and *b* are the largest perpendicular diameters measured and *c* is the sum of slices multiplied with thickness on CT or diffusion-weighted imaging on MRI). An additional CT scan was performed to determine brain edema or hemorrhagic transformation whenever patients had neurological deterioration. Assessment of brain CT or MRI was conducted by a neuroradiologist who was blinded to clinical picture.

For each patient, stroke severity was assessed using National Institutes of Health Stroke Scale (NIHSS) by a member of stroke team on admission, and subsequently on a daily during period of hospitalization. Age, sex, onset to admission time, baseline systolic and diastolic blood pressure were recorded. Vascular risk factors were investigated. Serum glucose, triglycerides (TG), total plasma cholesterol (TC), and low-density lipoprotein cholesterol (LDL-C) were measured. Dyslipidemia was defined as TG > 180 mg/dL, TC > 200 mg/dL or use of lipid-lowering medication [19]. Stroke subtypes were classified as large-artery atherosclerosis, cardioembolism, stroke of other determined etiology, and stroke of undetermined etiology according to the subtype classification criteria [25]. All enrolled patients received standard therapies based on standard guidelines [9].

### Measurement of plasma 20-HETE, EETs and DiHETEs levels

Whole blood (4 ml) was drawn from each patient on admission. Plasma was isolated following centrifugation and samples were stored at − 80 °C until analysis. Total plasma EETs and DiHETEs levels were measured using a stable isotope dilution GC/MS following base hydrolysis and separation on high performance liquid chromatography (HPLC), and plasma 20-HETE level was analyzed using a stable isotope dilution gas chromatography/mass spectrometer (GC/MS), as described in our previous studies [19, 20].

### Assessment of m-MCAI and clinical outcome

The primary outcome of this study was m-MCAI. The m-MCAI was diagnosed according to the following criteria [13, 26]: massive MCAI showed on follow-up CT including more than two-thirds space-occupying MCAI with midline shift and compression of the basal cisterns, and further consciousness status declined at least 1 point of consciousness item described in the NIHSS compared with the baseline consciousness status on admission, or deterioration of neurological status with clinical signs of uncal herniation and mass effect leading to early death or DHC.

All patients were followed up at 3 months after stroke by a certified stroke team member using questionnaires via telephone interview. The secondary outcome was vascular death and modified Rankin Scale (mRS) at 3 months. Vascular death was defined as vascular mortality due to coronary heart disease, ischemic stroke, or other vascular causes. A favorable outcome was considered as mRS ≤ 2 points, while mRS > 2 points was defined as a unfavorable outcome [14, 19–21].

### Statistical analysis

Previous studies have shown prevalence of m-MCAI is approximately 30 - 50% in patients with massive MCAI [2–4]. According to this estimate, we expected a minimum sample size requirement of 250 patients with massive MCAI for determining the difference in prevalence of m-MCAI within ±15% with 95% confidence intervals (CI) [20, 27].

The results are expressed as percentages for categorical variables, and continuous variables are expressed as mean ± Standard Deviation. Baseline and clinical characteristics were compared using χ^2^ test or Fisher exact test (categorical variables) and the Student *t* test (continuous variables) between patients with and without m-MCAI. We calculated 20-HETE by quartiles of increasing levels to evaluate for possible threshold effects for m-MCAI.

Multiple logistic regression analysis was used to assess the possible contributing factors for m-MCAI using variables with *P* values < 0.05 in univariate analysis, and reported as odds ratio (OR) with the 95% confidence intervals (CI). Furthermore, Cox proportional hazard model was performed to account for the probability of m-MCAI according to the 20-HETE levels levels, and reported using Kaplan-Maier curve.

All statistical analyses were performed using SPSS 16.0 (SPSS Inc., Chicago, IL, USA). All tests were two sided, and the threshold level of *P* < 0.05 was defined as statistical significance.

## Results

We prospectively registered 1542 patients with ischemic stroke who were admitted within 48 h of the onset of stroke between October 2011 and September 2014. Among the 1542 patients, 256 patients were massive MCAI. Seventy-seven of the 256 patients (30.1%) developed m-MCAI (60 of 77 patients [77.9%] occurred in the first 72 h, all occurred within 7 days after admission), 179 patients (69.9%) did not experience m-MCAI. Baseline clinical characteristics in patients with and without m-MCAI were presented in Table 1. Compared with patients without m-MCAI, younger age, atrial fibrillation, left MCAI, and subtype of cardio-embolism were significantly more frequent in patients with m-MCAI. For in-hospital management, patients with m-MCAI more frequently used mechanical ventilation (35.1% vs. 1.7%, *P* < 0.001) and decompressive surgery (27.2% vs. 1.7%, *P* < 0.001) than patients without m-MCAI (Table 1).

**Table 1:** Baseline clinical characteristics in patients with and without m-MCAI

Characteristics	Patients with m-MCAI (*n* = 77)	Patients without m-MCAI (*n* = 179)	*P* value
Age (years)	58.9 ± 14.2	66.7 ± 15.5	< 0.001
Men (n, %)	36 (46.8)	87 (48.6)	0.786
>60 y of age (n,%)	32 (41.6)	108 (60.3)	< 0.001
Hypertension (n, %)	35 (45.5)	94 (52.5)	0.301
Diabetes mellitus (n, %)	16 (20.8)	37 (20.7)	0.987
Atrial fibrillation (n, %)	45 (58.4)	66 (36.9)	0.002
Current smoker (n, %)	19 (24.7)	39 (21.8)	0.637
Dyslipidemia (n, %)	12 (15.6)	35 (19.6)	0.482
Plasma glucose (mmol/L)	7.7 ± 3.2	7.9 ± 3.5	0.652
Onset to admission time (h)	21.3 ± 8.9	23.2 ± 10.2	0.151
NIHSS score on admission	16.1 ± 7.3	14.9 ± 7.9	0.147
SBP on admission (mm Hg)	146.7 ± 26.4	144.7 ± 25.2	0.578
DBP on admission (mm Hg)	84.8 ± 14.7	85.3 ± 15.6	0.712
Temperature on admission, °C	36.9 ± 1.5	36.7 ± 1.7	0.349
Left MCA infarction (n, %)	49 (63.6)	78 (43.6)	0.004
Etiology (n, %)			0.014
Large-artery atherosclerosis	16 (20.8)	43 (24.0)	
Cardioembolism	49 (63.6)	74 (41.3)	
Other determined etiology	2 (2.6)	12 (6.7)	
Undetermined etiology	10 (13.0)	50 (27.9)	
Mechanical ventilation (n, %)	27 (35.1)	3 (1.7)	< 0.001
Decompressive craniectomy (n, %)	21 (27.2)	3 (1.7)	< 0.001
Outcome (n, %)			
Early neurological deterioration	77 (100.0)	47 (26.3)	< 0.001
Mortality during 3 months	60 (77.9)	75 (41.9)	< 0.001
mRS > 2 points at 3 months	74 (96.1)	128 (71.5)	< 0.001

*m-MCAI* malignant middle cerebral artery infarction, *MCA* middle cerebral artery; *NIHSS* National Institutes of Health Stroke Scale, *SBP* systolic blood pressure, *DBP* diastolic blood pressure, *tPA* tissue plasminogen activator, *mRS* modified Rankin Scale

Unfavorable outcome (mRS > 2) was more frequent in patients with m-MCAI than those without m-MCAI. Sixty of patients (77.9%) with m-MCAI died within 3 months after stroke, and only 3 (3.9%) of the survivors were independent (mRS ≤2) at 3 months. Among the patients without m-MCAI, 75 (41.9%) patients were dead during 3 months, and 51 (25.5%) were independent at 3 months (Table 1).

Early signs of cerebral infarction in the first CT scan were detected more frequently in patients who later developed m-MCAI than those did not experience m-MCAI (89.6% vs. 77.8%; *P* = 0.045, Table 2). Severe mass effect (grade II and III) and extent of MCAI (ASPECTS) on CT scan at admission, and final infarct volume > 145 cm^3^ at second CT scan were significantly more frequent in m-MCAI group than non m-MCAI group (Table 2).

**Table 2:** Neuroimaging finding and plasma CYP450 metabolite levels on admission in patients with and without m-MCAI

Factor	Patients with m-MCAI (*n* = 77)	Patients without m-MCAI (*n* = 179)	*P* value
Early signs at the first CT scan (n, %)	69 (89.6)	141 (78.8)	0.045
Hypodensity	59 (76.6)	139 (77.7)	0.863
Mass effect	56 (72.7)	91 (50.8)	< 0.001
Grade I	15 (19.5)	80 (44.7)	
Grade II	27 (35.1)	11 (6.1)	
Grade III	14 (18.2)	0 (0.0)	
ASPECTS	4 (3-6)	7 (6-10)	0.036
Infarct volume > 145 cm^3^ at second CT scan (n, %)	71 (92.2)	18 (10.1)	< 0.001
20-HETE at admission (pmol/L)	1924.6 ± 194.8	1698.7 ± 173.5	< 0.001
EETs at admission (nmol/l)	66.7 ± 17.6	70.4 ± 18.9	0.123
DiHETEs at admission (nmol/l)	80.2 ± 19.1	75.8 ± 17.6	0.089

*m-MCAI* malignant middle cerebral artery infarction, *CT* computed tomography, *ASPECTS* Alberta Stroke Program Early CT Score, *CYP450* cytochrome P450, *HETE* hydroxyeicosatetraenoic acid, *DiHETEs* dihydroxyeicosatrienoic acids, *EET* epoxyeicosatrienoic acids

The mean 20-HETE level was 1924.6 ± 194.8 pmol/L in patients with m-MCAI, and 1698.7 ± 173.5 in patients without m-MCAI on admission (*P* < 0.001, Table 2). There were no significant differences of EETs and DiHETEs levels between two groups (*P* > 0.05, Table 2). The mean 20-HETE level was 1789 ± 174.6 pmol/L in 256 patients with massive MCAI, with quartile levels as follows: 826 to 1456 pmol/L (first quartile, *n* = 48); 1457 to 1768 pmol/L (second quartile *n* = 78); 1769 to 2024 pmol/L (third quartile, *n* = 76); and 2025 to 2737 pmol/L (fourth quartile, *n* = 54). The incidence of m-MCAI increased with increasing quartile levels of 20-HETE (0% [0/48], 14.1% [11/78], 35.5% [27/76], and 72.2% [39/54] in patients from first quartile to fourth quartile, respectively, *P* < 0.001). The median time of developed m-MCAI in patients with massive MCAI was 4 days after admission for low 20-HETE levels, 32 h for intermediated 20-HETE levels, and 18 h for high 20-HETE levels.

**Fig. 2:**
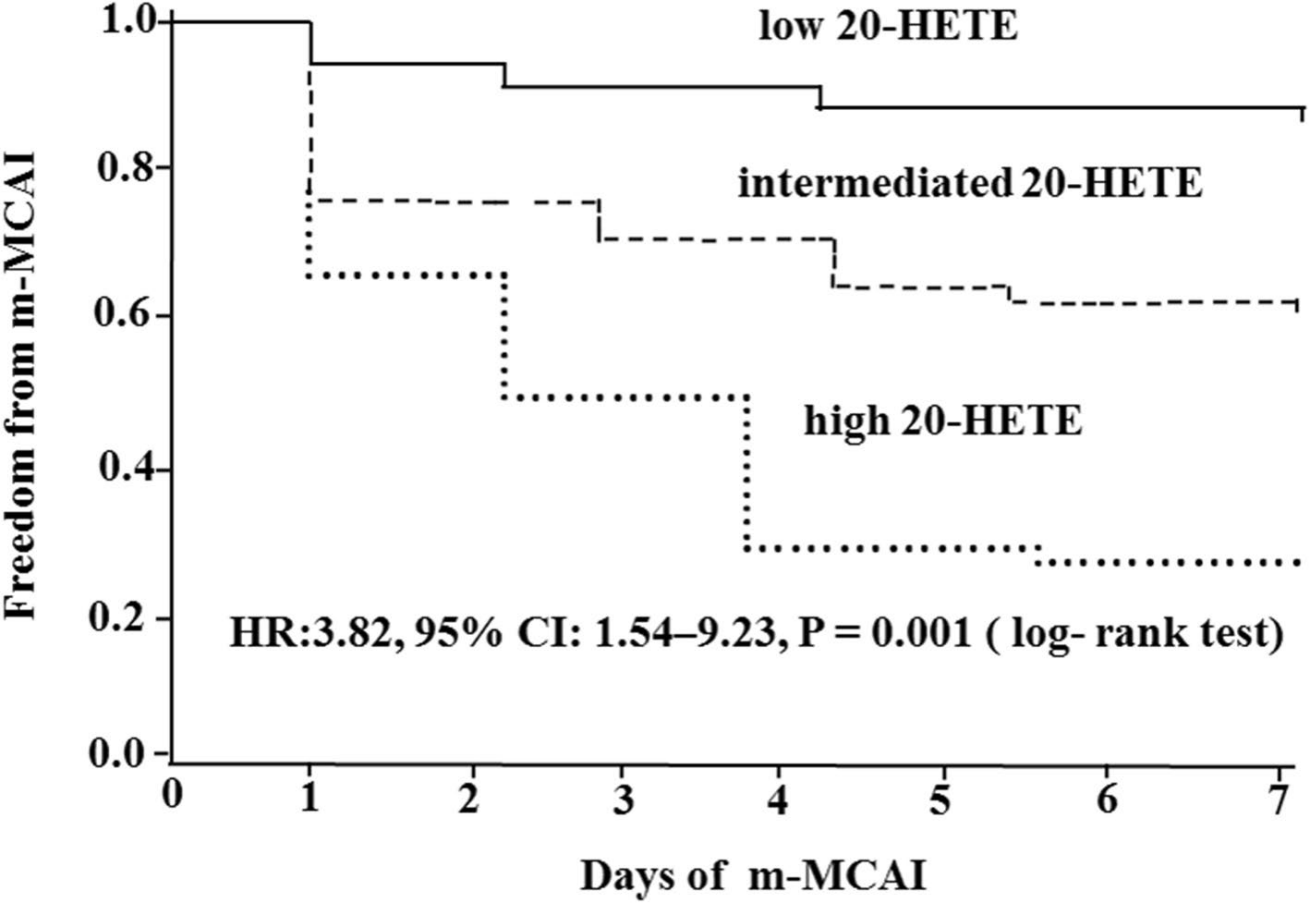
Probability of survival free of m-MCAI. Kaplan-Maier analysis of cumulative freedom from m-MCAI associated with HETE. m-MCAI indicates malignant middle cerebral artery infarction; HETE indicates hydroxyeicosatetraenoic acid

20-HETE levels and final infarct volume > 145 cm^3^ were found to be independent predictors of m-MCAI after adjusting the covariates (Table 3). The odds ratio for m-MCAI increased with increase in quartiles of 20-HETE level, using the first quartile as the reference, the third and fourth quartiles of 20-HETE levels were found to be independent predictors of m-MCAI (OR: 2.86; 95% CI: 1.16 – 6.68; *P =* 0.025, and OR: 4.23; 95% CI: 1.35 – 8.26; *P =* 0.002, respectively, Table 3). Cox proportional hazard curve is showed in the Fig. 2, there was an increase in the risk of m-MCAI with higher level of 20-HETE (HR: 3.82, 95% CI: 1.54 – 9.23, *P* = 0.001 (log- rank test).

**Table 3:** Predictors of m-MCAI and odds ratio according to 20-HETE quartiles

Factor	OR	95% CI	*P* value
20-HETE Quartile, pmol/L			
Quartile 1 (reference)			
Quartile 2	1.23	0.54 – 2.96	0.425
Quartile 3	2.86	1.16 – 6.68	0.025
Quartile 4	4.23	1.35 – 8.26	0.002
Age > 60 y	0.94	0.83 – 1.64	0.532
Atrial fibrillation	1.33	0.75 – 2.04	0.342
Left MCA infarction	1.42	0.96 – 3.87	0.126
Cardioembolism	1.33	0.97 – 3.58	0.113
Early signs of CT	1.21	0.89 – 2.21	0.325
Mass effect (Grade II and III)	1.38	0.98 – 4.36	0.063
ASPECTS ≤4	1.22	0.99 – 3.96	0.083
Final infarct volume > 145 cm^3^	1.67	1.46 – 6.25	0.026

*m-MCAI* malignant middle cerebral artery infarction, *MCA* middle cerebral artery; *HETE* hydroxyeicosatetraenoic acid, *CI* confidence intervals, *OR* odds ratio, *CT* computed tomography, *ASPECTS* Alberta Stroke Program Early CT Score

## Discussion

The present results showed that the incidence of m-MCAI was very high in patients with massive MCAI. 20-HETE level was independent predictor of m-MCAI, the odds ratio for m-MCAI increased with increase in quartiles of 20-HETE levels, this may be of use in therapeutic decision making and the timing of DHC [28].

In recent decades, clinical risk factors, neuroimaging variables, and molecular markers have been thoroughly investigated for prediction of m-MCAI [2–4, 8, 13, 14]. However, the sensibility and specificity of these markers for prediction of m-MCAI are insufficient. Some studies revealed that the sensitivity of brain CT scan was high, while the specificity was low for identifying m-MCAI [23, 29]. In present study, we found that prevalence of early signs of cerebral infarction and mass effect (grade II and grade III) was more prevalent, and ASPECTS was significantly lower on the first CT scan in patients who later developed m-MCAI than those did not experience m-MCAI. However, these neuroimaging variables were not independent predictor of m-MCAI. Our results were in accordance with other previous studies [5, 8, 23, 29], indicating that clinical factors were not sufficient to identify patients with impending malignant brain edema and m-MCAI [2, 14]. In recent study, promising results have been obtained from neuroimaging tests such as single-photon emission CT and diffusion-weighted MRI in the prediction of m-MCAI [30]. Although these techniques are most reliable predictor for m-MCAI quickly and accurately, they are unable to directly predict the development of massive brain edema.

The underlying basic mechanisms of m-MCAI are not completely understood. The loss of integrity of the endothelial basal lamina and BBB disruption play key roles in pathophysiological mechanisms of malignant edema formation after massive MCAI. In this study, we found that elevated plasma 20-HETE may be as an independent predictor for m-MCAI in patients with acute massive MCAI, and this is the first to identify a positive relationship between 20-HETE levels and m-MCAI.

20-HETE, as a potent vasoconstrictor, is associated with cerebral ischemia injury, cerebral edema, and unfavorable outcomes after subarachnoid hemorrhage [31]. The increase of vascular permeability and subsequent extravasation of serum components is one of principal causes in development of brain edema after ischemic stroke. 20-HETE can regulate cerebral vascular tone, constrict cerebral arteries, and increase vascular permeability by activating intracellular protein kinase C signaling pathway [32], which is involved in apoptosis or cell death [33]. Neuronal apoptosis is an important mechanism of brain ischemic damage in animal experiments [34], and is associated with mortality and poor functional prognosis after ischemic stroke [35]. 20-HETE may also injure vascular smooth muscle cells and endothelial cells by inhibition of Na^+^, K^+^ − ATPase activity and large-conductance Ca^2+^ − sensitive K^+^ channel, and increase of Ca^2+^ influx via L-type Ca2^+^ channels [36]. In addition, 20-HETE promotes formation of oxygen radicals, which may cause endothelial injury [37, 38]. Inhibitor of 20-HETE can reverse the decrease of cerebral blood flow following subarachnoid hemorrhage and reduce infarct size after transient cerebral ischemia in animal experiments [39, 40]. All of these previous studies suggest a potential molecular mechanism of 20-HETE might lead to m-MCAI after acute massive MCAI.

Our previous study showed that low EETs levels and high DiHETEs levels were associated with early neurological deterioration in minor ischemic stroke (NIHSS score ≤ 3) [20]. However, we did not find such association for m-MCAI in this study. In present study, we enrolled patients with acute massive MCAI, NIHSS scores on admission were higher, standard deviations of NIHSS, EETs levels and DiHETEs levels were bigger than patients with minor ischemic stroke. Furthermore, the small samples were also another reason of no association between low EETs levels and m-MCAI. Thus, larger samples studies are necessary to validate our present findings in future.

Despite our findings are interesting, several limitations of this study should be noted. First, this was a two-center study, the samples were small. Therefore, our current findings should be validated in larger samples, multi-center studies. Second, plasma CYP metabolite levels may dynamic changes after ischemic stroke. Blood samples for CYP metabolites were drawn from each patient at admission. We did not measure plasma CYP metabolite levels during follow-up in non-surviving and surviving patients. Third, m-MCAI could be better detected if brain CT scan were consistently undertaken earlier, or repeated every 48 h. However, brain CT scan was performed at admission and repeated between 3 days and 7 days of hospitalization or when patients had neurological deterioration in this study. Therefore, further well designed studies are needed to confirm our current findings.

## Conclusion

The incidence of m-MCAI was very high in patients with massive MCAI, the prognosis of m-MCAI is very poor. Elevated plasma 20-HETE may be as a predictor for m-MCAI. Our findings may be useful in clinical practice in therapeutic decision making and the timing of DHC.

## Data Availability

The datasets used and/or analysed during the current study are available from the corresponding author on reasonable request.

## Abbreviations

MCAI: Massive middle cerebral artery infarction
m-MCAI: Malignant MCAI
DHC: Decompressive hemicraniectomy
20-HETE: 20-hydroxyeicosatetraenoic acid
CT: Computed tomography
ASPECTS: Alberta Stroke Program Early CT Score
BBB: Blood-brain barrier
CYP: Cytochrome P450
EETs: Epoxyeicosatrienoic acids
sEH: Soluble epoxide hydrolase
MRI: Magnetic resonance imaging
mRS: Modified Rankin scale
TG: Triglycerides
TC: Total plasma cholesterol
LDL-C: Low-density lipoprotein cholesterol
CI: Confidence intervals
OR: Odds ratio
